# Exposure to Normobaric Hypoxia Combined with a Mixed Diet Contributes to Improvement in Lipid Profile in Trained Cyclists

**DOI:** 10.3390/nu13103481

**Published:** 2021-09-30

**Authors:** Kamila Płoszczyca, Miłosz Czuba, Józef Langfort, Marcin Baranowski

**Affiliations:** 1 Department of Kinesiology, Institute of Sport, 01-982 Warsaw, Poland; kamila.ploszczyca@insp.waw.pl; 2 Department of Applied and Clinical Physiology, Collegium Medicum University of Zielona Gora, 65-417 Zielona Góra, Poland; 3 Department of Sports Theory, The Jerzy Kukuczka Academy of Physical Education, 40-065 Katowice, Poland; langfort@cmdik.pan.pl; 4 Department of Physiology, Medical University of Bialystok, 15-222 Bialystok, Poland; marcin.baranowski@umb.edu.pl

**Keywords:** hypoxia, intermittent hypoxic training, lipid profile, mixed diet, athletes

## Abstract

This study aimed to analyze the effects of live high-train low method (LH-TL) and intermittent hypoxic training (IHT) with a controlled mixed diet on lipid profile in cyclists. Thirty trained male cyclists at a national level with at least six years of training experience participated in the study. The LH-TL group was exposed to hypoxia (FiO_2_ = 16.5%) for 11–12 h a day and trained under normoxia for 3 weeks. In the IHT group, participants followed the IHT routine three times a week under hypoxia (FiO_2_ = 16.5%) at lactate threshold intensity. The control group (N) lived and trained under normoxia. The results showed that the 3-week LH-TL method significantly improved all lipid profile variables. The LH-TL group showed a significant increase in HDL-C by 9.0% and a decrease in total cholesterol (TC) by 9.2%, LDL-C by 18.2%, and triglycerides (TG) by 27.6%. There were no significant changes in lipid profiles in the IHT and N groups. ∆TG and ∆TC were significantly higher in the LH-TL group compared to the N group. In conclusion, hypoxic conditions combined with a mixed diet can induce beneficial changes in lipid profile even in highly trained athletes. The effectiveness of the hypoxic stimulus is closely related to the hypoxic training method.

## 1. Introduction

Exposure of the human body to hypoxic conditions, both at rest and in combination with physical exercise, contributes to the activation of numerous adaptive mechanisms. With these adaptive changes, an increase in the effectiveness of conventional training methods is observed, resulting in improvements in athletes’ exercise capacity [1,2]. In recent years, much attention has been paid to the concept of using a hypoxic environment as a therapeutic agent to support the treatment and prevention of non-communicable diseases and to improve the quality of life of patients and older adults [3].

Blood lipid profile is one of the indicators of cardiovascular disease (CVD) risk. Increases in blood levels of triglycerides (TG) and LDL cholesterol (LDL-C) and decreases in HDL cholesterol (HDL-C) are associated with an elevated risk of CVD [4]. Most studies have indicated that lipid ratios, such as Castelli’s risk index I and II (CRI-I and CRI-II) and the atherogenic index of plasma (AIP), may be more accurate predictors of cardiovascular risk than traditional lipid indicators that may remain in the normal range [5,6,7].

Regular physical activity improves the blood lipid profile [8] and atherogenic indices [9]. The range of adaptations depends on the type, frequency, intensity, and duration of exercise [10,11,12]. Athletes have higher HDL-C, lower TG, and lower total cholesterol (TC) and LDL-C than sedentary individuals [12,13,14,15,16,17]. There are also reports that did not confirm that the lipid profile of athletes differed significantly from non-athletes [18,19]. Furthermore, it worth noting that despite regular training, the values of lipid profile indices may exceed the reference range [15,20,21]. This largely depends on diet, gender, body fat mass, and the type of sport [12,18,22]. It has been shown that athletes with elevated LDL-C levels may be at higher risk for atherosclerosis and CVD [23]. This is because intense physical exercise elevates the rate of oxygen consumption, resulting in the formation of reactive oxygen species (ROS), while low-density lipoproteins (LDL) are highly susceptible to free radical-mediated oxidative modification [24].

The lipid profile of athletes can be altered by diet, supplementation [22,25,26], and rest periods during the training process [27,28]. Furthermore, environmental conditions may also affect lipid profile and CVD risk [29]. For example, populations living at high altitudes have lower CVD risk and are characterized by lower mortality [30]. Some studies have revealed that high-altitude natives have lower TC and LDL-C and higher HDL-C levels [31,32,33]. An improvement in the lipid profile was observed in participants of high-mountain expeditions [34,35], which indicates that even short stays at high altitudes (above 4000 m) may lead to beneficial health effects. It is not known, however, whether similar changes in blood occur in athletes living and training in normobaric hypoxia corresponding to moderate altitudes (2000–3000 m).

Currently, the most common variants of altitude/hypoxic training include the live high-train low (LH-TL) method and intermittent hypoxic training (IHT) [1,36,37]. The findings of recent studies indicate that IHT training performed by obese individuals enhances body fat reduction and significantly improves lipid profile [38,39,40]. Despite these reports, studies on the effects of LH-TL and IHT on the lipid profile of athletes are still lacking. Previous studies in this field have mainly focused on the efficacy of the IHT procedure only in non-athletes [41,42,43,44,45,46,47]. In our recent study [48], we found no changes in the lipid profile of athletes after short-term, six-day, passive, intermittent hypoxic exposure (IHE—6 doses of 3–8-min periods of hypoxia at FiO_2_ of 14–12% interrupted by 3–5-min periods of normoxia and repeated for 60–80 min). However, it remains unclear whether the use of hypoxia during prolonged passive exposure (LH-TL; 10–12 h per day) and training (IHT) can alter the lipid profile in individuals with high levels of training adaptation.

We hypothesize that the combination of hypoxia and physical exercise may contribute to beneficial changes in lipid profile and atherogenic indices in athletes. Therefore, the aim of this study was to analyze the effects of LH-TL and IHT training with a mixed diet on lipid profile and values of atherogenic indices (CRI-I, CRI-II, and AIP) in trained cyclists.

## 2. Materials and Methods

### 2.1. Study Participants

The study examined thirty trained male cyclists at a national level. The basic inclusion criteria were a minimum of six years of training experience and at least a six-month washout period from previous altitude training. All athletes had current medical examinations, without any contraindications to performing exhaustive exercise in a hypoxic environment. The participants provided their written voluntary informed consent before the participation. Study participants were randomized to three groups: two experimental groups and a control group. During the experiment, one participant from the control group withdrew from the study due to an injury. The first experimental group (LH-TL) (*n* = 10; age: 22.9 ± 6.3 years; body height 1.83 ± 0.08 m; body mass: 70.4 ± 7.1 kg; body fat content: 6.9 ± 2.1%; fat free mass: 65.5 ± 6.0 kg; training experience: 6.5 ± 1.3 years) was exposed to normobaric hypoxia (FiO_2_ = 16.5%, ~2000 m) at rest and during sleep for 11 to 12 h a day. Training in this group was performed under normoxia. In the second experimental group (IHT) (*n* = 10; age: 26.4 ± 8.1 years; body height 1.82 ± 0.08 m; body mass: 70.8 ± 9.4 kg; body fat content: 10.4 ± 2.6%; fat-free mass: 63.5 ± 8.9 kg; training experience: 6.1 ± 1.1 years), participants followed an IHT routine three times a week under normobaric hypoxia (FiO_2_ = 16.5%, ~2000 m). The control group (N) (*n* = 9; age: 29.1 ± 6.8 years; body height 1.81 ± 0.03 m; body mass: 69.9 ± 5.9 kg; body fat content: 7.3 ± 1.2%; fat-free mass: 64.4 ± 6.2 kg; training experience: 6.6 ± 1.7 years) lived and trained under normoxic conditions. Lipid profiles were not taken into consideration during the subjects’ selection. 

The research project was conducted according to the Helsinki Declaration and was approved (No. R-I-002/325/2019) by the Bioethics Committee of the Medical University of Bialystok, Poland.

### 2.2. Study Design

The evaluation included two research series (S1, S2). All the tests were performed during the pre-competitive period. Between S1 and S2, athletes from all groups followed a similar training program for 3 weeks. The only factor that differentiated the training protocols between groups was the exposure of the IHT group to normobaric hypoxia (FiO_2_ = 16.5%, ~2000 m) in a hypoxic chamber (AirZone 40, Air Sport, Poland). The participants from the LH-TL and N groups followed the same training routine as the IHT group but under normoxic conditions. In addition, in the LH-TL group, participants spent 11–12 h a day (evenings and nights) under normobaric hypoxia (FiO_2_ = 16.5%) in a hypoxic training center (Air Zone, Warsaw, Poland).

### 2.3. Training Program

The training program included three microcycles (3 weeks) with progressive training loads. All the groups followed the same training routines with individually adjusted intensity zones (Table 1). Each training session in the laboratory (T1,T2,T3; three times per week) included a 15 min warm-up, 30 to 40 min main part, and 15 min cool-down. Intensity during these sessions was adjusted individually to each study participant based on the LT workload determined under normoxia (WR_LT_; LH-TL, N groups) or under hypoxia (WR_LThyp_; IHT group). The warm-up during all laboratory sessions was performed using the intensity at the level of 65–70% WR_LT_/WR_LThyp_. In the main part, the intensity was increased to 100% WR_LT_/WR_LThyp_. This level of intensity was maintained for 30 min (first week), 35 min (second week), and 40 min (third week). The cool-down included 15 min of continuous exercise with an intensity of 65–70% WR_LT_/WR_LThyp_. After completion of the final part of the laboratory session, cyclists performed a two-hour ride under normoxic conditions at an intensity of 60–75% WR_LT_.

### 2.4. Measurements during the Experiment 

During the experiment, each research series (S1 and S2) was performed after an overnight fast and started by drawing venous blood (10 mL) from the antecubital vein to determine total cholesterol (TC), high-density lipoprotein cholesterol (HDL-C), and triglycerides (TG) (Cobas 6000/c501, Roche, Germany). The low-density lipoprotein cholesterol (LDL-C) was calculated using the Friedewald formula: LDL-C = TC– (TG/5+HDL-C) (Friedewald et al. 1972). Furthermore, the atherogenic index (AIP) and lipid ratios (CRI-I, CRI-II) were calculated using the following formulas [7,49]:

Castelli’s risk index I (CRI-I) = TC/HDL-C
Castelli’s risk index II (CRI-II) = LDL-C/HDL-C
Atherogenic index of plasma (AIP) = log_10_(TG/HDL-C)

After obtaining the blood samples, body height, body mass, and body composition were also measured (InBody 220, Biospace, Korea). Next, two hours after a light mixed meal (5 kcal/L kg of body mass, 50% CHO, 30% Fat, 20% Pro), study participants performed a graded exercise test (40 W/3 min) using the Excalibur Sport cycle ergometer (Lode, Netherlands) in order to measure VO_2max_ (MetaLyzer 3B-2R, Cortex, Germany) and lactate threshold workload (WR_LT_). These data were used to determine an individual training workload for the experiments.

Furthermore, after 48 h of rest during S1, the IHT group performed the same exercise test on the cycle ergometer under normobaric hypoxia conditions in order to determine the individual training load for the IHT workouts (WR_LThyp_).

### 2.5. Diets during the Experiment

Throughout the experiment, all athletes lived at the same accommodation and followed the same training schedule, sleeping time, and diet. During the experiments, the participants consumed a controlled mixed diet (50% CHO, 20% Fat, 30% Pro; Table 2). Daily energy intake was set at ~3500 kcal. Athletes did not take any nutrition supplements. 

### 2.6. Statistical Analysis

The data were analyzed using StatSoft Statistica 13.3 software. The results were presented as arithmetic means (x) ±standard deviations (SD). The statistical significance was set at *p* < 0.05. Prior to all statistical analyses, the normality of the distribution of variables was checked using the Shapiro–Wilk test. The paired samples *t*-test was used to determine the significance of differences in lipid profile before and after training within groups. Comparison between groups was performed using analysis of covariance (ANCOVA), and the baseline levels of the dependent variables were used as covariates. The post-hoc Tukey test was used for significant differences. Effect sizes (ESs) were calculated from standardized differences (Cohen’s *d* values). Threshold values for Cohen ES statistics were considered to be small (0.20–0.60), moderate (0.60–1.20), large (1.20–2.0), very large (2.0–4.0), or extremely large (>4.0) [50].

## 3. Result

**Figure 1:**
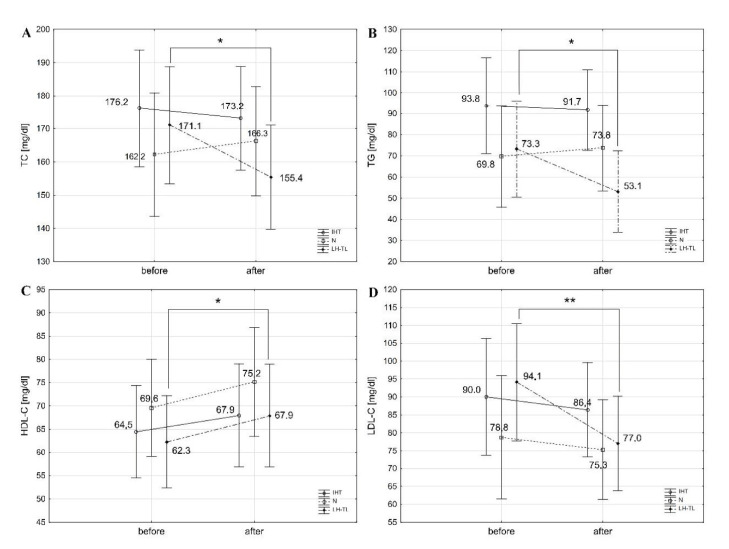
Lipid profile in cyclists before (S1) and after training (S2) in LH-TL, IHT, and the control group (N). Abbreviations: TC, total cholesterol (**A**); TG, triglycerides (**B**); HDL-C, high-density lipoprotein cholesterol (**C**); LDL-C, low-density lipoprotein cholesterol (**D**). * *p* < 0.05, ** *p* < 0.01 before vs. after training.

The LH-TL group showed a statistically significant increase in HDL-C by 9.0% (t = −2.40, *p* < 0.05; d = 0.47) and a decrease in TC by 9.2% (t = 3.20, *p* < 0.05; d = 0.61), LDL-C by 18.2% (t = 3.55, *p* < 0.01; d = 0.88), and TG by 27.6% (t = 2.67, *p* < 0.05; d = 0.88) (Figure 1). There was also a statistically significant improvement in atherogenic indices: AIP (t = 2.77, *p* < 0.05; d = 0.88), CRI-I (t = 3.81, *p* < 0.01; d = 0.96), and CRI-II (t = 3.93, *p* < 0.01; d = 0.96) (Table 3). There were no statistically significant changes in lipid profile or atherogenic indices in the IHT group and the control group (N).

There was a significant effect of group on ∆TG after controlling for the effect of baseline level of TG (F = 4.64, *p* < 0.05). Additionally, the analysis showed an effect of group on ∆TC and ∆AIP slightly above the accepted level of significance (F = 2.81, *p* < 0.08; F = 2.91, *p* < 0.08, respectively). ∆TG and ∆TC were significantly (*p* < 0.05) higher in the LH-TL group compared to the N group (Table 4). ∆AIP was also higher in the LH-TL compared to the N group, although the difference was not significant (*p* < 0.08) (Table 5). The covariates (baseline levels) were significantly related to ∆TC (F = 8.46, *p* < 0.01), ∆LDL-C (F = 13.9, *p* < 0.001), ∆TG (F = 10.89, *p* < 0.01), ∆CRI-I (F = 8.04, *p* < 0.01), and ∆CRI-II (F = 14.70, *p* < 0.001). When the baseline levels of TC, LDL-C, TG, CRI-I, and CRI-II were higher, the decrease in these variables after the intervention was also higher.

Body mass and body composition did not change significantly with training in any group (Table 6).

## 4. Discussion

To the authors’ knowledge, this is the first study to examine the effects of LH-TL training and IHT with a controlled mixed diet on lipid profile and atherogenic indices in athletes. The results of our study showed that a three-week LH-TL protocol (2000 m, 11–12 h per day) resulted in a significant improvement in all lipid profile variables analyzed and atherogenic indices. These changes were not observed in the IHT group (3 weeks, 2000 m, 3 continuous training sessions with lactate threshold intensity/week) and in the control group of cyclists training and living in normoxia. 

It is common that physical activity leads to an improved lipid profile [8], while athletes tend to have higher HDL-C levels and lower TC, LDL-C, and TG values than non-athletes [14,15,16,17,51]. However, the results of previous studies on changes in lipid profile of athletes induced by training programs are divergent [23,52,53]. Factors that may explain these discrepancies include the sports level of the participants, diet, training intensity, and volume. The current level of training adaptation may also play a key role [28,54,55,56]. Furthermore, the phenomenon of hypoxia, which is commonly used in the training process, can also have a significant impact on the lipid profile of athletes. The basis for the above hypothesis is the observed adaptive changes occurring in high-altitude populations [31,32,33] and participants of high-altitude expeditions [34,35,57]. It has been observed that blood levels of TC, LDL-C, and TG are reduced following prolonged exposure to high altitude [31,35,57,58], while HDL-C levels increase [31,32]. Similar changes were also reported after living at moderate altitudes (days–weeks), where a decrease in TC and LDL-C levels [59,60,61] and an increase in HDL-C levels [61] were observed. In a recent study by Gao et al. [62], the authors did not find statistically significant changes in the lipid profile following a four-week LH-TL protocol at a simulated altitude of 2300 m. It should be noted that these studies were performed with nontrained individuals and those with obesity and metabolic syndrome. A study involving athletes was conducted by Pialoux et al. [63]. After a two-week LH-TL protocol at 2500 m to 3500 m, a mixed group of cross-country skiers (males and females) showed no change in TC and TG levels. The results of this study are in contrast to the results of the present research, which demonstrated a significant improvement in lipid profile following three weeks of the LH-TL protocol at 2000-m altitude in cyclists. The changes in TG and TC were significantly higher after LH-TL training compared to training in normoxia. Despite using a controlled mixed diet, Pialoux et al. [63] examined a small mixed sample (males and females), which may have significantly affected the TC and TG results obtained. It is worth noting that the lipid profile is altered during the menstrual cycle [64], which was not reported in the study. Furthermore, the HDL and LDL cholesterol fractions were not recorded.

The mechanisms responsible for the improvement of the lipid profile induced by hypoxia have not yet been fully explained. Tin’kov and Aksenov [65] suggested that reduction in plasma TC concentration due to hypoxic exposure may be induced by the increase in cholesterol 7α-hydroxylase (CYP7A1) activity. CYP7A1 is a rate-limiting enzyme used in the synthesis of bile acid from cholesterol. The conversion of cholesterol to bile acids contributes to the elimination of TC from the body; thus, the increase in activity of CYP7A1 may be beneficial in subjects with hypercholesterolemia and CVD [66]. However, recent studies indicated that CYP7A1 activity and bile acid synthesis are downregulated under hypoxic conditions [67,68], which is likely to exclude this mechanism as responsible for the decrease in TC plasma levels following the use of the LH-TL method. Reduction of blood TC after LH-TL training occurred mainly due to the decrease of its low-density fractions. Potentially, the decrease of LDL-C may be caused by an increase in the uptake of LDL particles from the blood into hepatocytes. LDL is cleared from the circulation via the LDL receptors located on the surface of hepatocytes. Adequate levels of LDL receptors are necessary to remove excess cholesterol-containing LDL [69]. Studies performed on an animal model showed that exercise training increase the hepatic expression of the LDL receptor [70,71]. However, it is unknown how altitude/hypoxic training affects LDL receptor expression. 

The mechanisms associated with the improvement of HDL-C after LH-TL are also unclear. In a recent study, Gao et al. [62] suggested that exposure to hypoxia may prevent the loss of HDL-C due to the increased expression of the lipoprotein lipase gene as a result of hypoxia-induced factor 1 (HIF1) upregulation. Furthermore, the activation of HIF1 may enhance HDL particle synthesis by stimulating the expression of ATP-binding cassette transporter ABCA1 [72,73]. Higher HDL-C levels may also be associated with greater concentrations of the less dense HDL2 subfraction and apoprotein A-I [74,75]. Further research is needed to elucidate the mechanisms of lipid profile improvement by a hypoxic stimulus. 

There are reports in the literature that not only prolonged exposure to hypoxia but also regular training sessions under these conditions have a beneficial effect on lipid profile. Haufe et al. [76] suggested that the interaction of exercise and hypoxia may have a much greater effect on blood TG levels compared to either stimulus alone. For example, IHT training performed at moderate altitudes (2000–3000 m) has been shown to lead to a decrease in blood TG levels [76,77]. The beneficial effect of hypoxic training on TG levels is explained by a higher rate of lipid oxidation due to increased expression of mRNA encoding peroxisome proliferator-activated receptor-gamma coactivator 1α (PGC-1α) protein, which induces mitochondrial biogenesis and plays a key role in regulating fatty acid oxidation in muscles [76,78,79]. However, the results to date on the effect of IHT training on improving blood lipid profile are inconclusive. Some studies indicate that IHT training improves lipid profile but does not increase the effects induced by normoxia training [41,47]. There are also reports that do not support a beneficial effect of IHT training on lipid profile [42,43,45]. It is worth noting that previous studies in which the lipid profile was not altered focused mainly on obese subjects who performed low-intensity exercise (55–60% VO_2max_) at 2000–2500 m [42,43,45]. In contrast, Debevec et al. [80] observed a decrease in TC levels and a reduction in LDL-C in physically active men after 10 days of an IHT protocol at an altitude of 4000 m. Similar changes were not observed in the group subjected to passive exposure to hypoxia. Similarly, Haufe et al. [76] demonstrated that a four-week IHT protocol (FiO_2_ = 15%, ~3000 m) reduced blood TG levels in healthy male non-athletes, which was not observed after training under normoxia. IHT training sessions were performed three times a week at an intensity around the lactate threshold. The findings were not confirmed by our results. After a training protocol similar to that used in the study of Haufe et al. [76], no significant changes were observed in the lipid profile of the cyclists in the IHT group (FiO_2_ = 16.5%, ~2000 m). 

We can presume that the intensity of the hypoxic stimulus may be a key factor in the beneficial effects of IHT on lipid profile. Furthermore, it is important to note that the intensity of hypoxia induced by IHT results from the combination of two components: altitude and exercise intensity. Wood et al. [81] reported that high-intensity interval training provides greater improvement in HDL-C compared to moderate-intensity continuous training. As mentioned above, one of the mechanisms responsible for the reduction in blood TG levels is the increased expression of mRNA encoding the PGC-1α protein [76,78,79]. In a recent study [82], the authors found that higher training intensity contributes to higher PGC-1α gene expression. We suspect that higher training intensity or a higher hypoxic stimulus may lead to an improvement in the lipid profile after IHT training. However, this issue requires further research. 

The best recognized environmental factor that can significantly modify the lipid profile is the diet. For example, a recent meta-analysis revealed that dietary fat manipulation has a significant influence on blood lipid levels in people with overweight or obesity without metabolic disturbances [83]. Results presented in another meta-analysis led to the conclusion that a low-carbohydrate diet has opposite effects on two important cardiovascular risk factors, i.e., it causes greater weight loss with a simultaneous increase of LDL cholesterol [84]. Moreover, even the low-energy mixed diet intervention can also change blood levels of lipoproteins [85]. It is reasonable to suppose that by purposeful selection of a balanced mixed diet, which participants consumed in the present study, we were able to minimize the influence of the applied diet on the fluctuation of lipid profile in our subjects. If so, our study also indicates that the observed beneficial changes in the lipid profile were caused by the training process under hypoxic conditions.

The results of this study indicate that the extent of changes in lipid variables is related to baseline values by the following rule: the higher the baseline level of TC, LDC-C, TG, CRI-I, and CRI-II, the greater their improvement in response to hypoxia. Some studies reveal that athletes who consume high-carbohydrate or high-fat diets increase their risk of developing hypercholesterolemia. In particular, this phenomenon was observed in keto-adapted endurance athletes [22] and athletes under statin therapy [86]. Thus, further research on the effect of the LH-TL and IHT method on the lipid profile should focus on well-trained athletes whose indices of lipid profile exceed the reference ranges. This approach allows verifying whether training under hypoxic conditions causes changes that are not stimulated by regular exercise.

## 5. Conclusions

Our findings indicate that the effectiveness of the hypoxic stimulus is closely related to the training method using the hypoxic environment, which determines the duration of exposure of the human body to hypoxia. Our results provide evidence of the beneficial effects of hypoxia rather than just exercise or diet on the lipid profile in trained individuals. The most beneficial changes in lipid profile were observed in the LH-TL group, which was passively exposed to hypoxia for the longest time (11 to 12 h a day for 3 weeks). Similar training with a controlled mixed diet in the control and IHT groups did not induce similar adaptations. Based on the results of this study, it can also be concluded that the magnitude of changes in the lipid profile after the hypoxic intervention will be greater in athletes with a disturbed lipid profile.

## Figures and Tables

**Table 1:** Training program during the experiment.

Day	Microcycle 1	Microcycle 2	Microcycle 3
1	T1 + 2 h endurance training (60–75% of WR_LT_)	T2 + 2 h endurance training (60–75% of WR_LT_)	T3 + 2 h endurance training (60–75% of WR_LT_)
2	3–4 h of endurance training 60–75% of WR_LT_ with high-speed intervals (2 × 6 × 10 s-max)	3–4 h of endurance training 60–75% of WR_LT_ with high-speed intervals (2 × 6 × 10 s-max)	3–4 h of endurance training 60–75% of WR_LT_ with high-speed intervals (2 × 6 × 10 s-max)
3	T1 + 2 h endurance training (60–75% of WR_LT_)	T2 + 2 h endurance training (60–75% of WR_LT_)	T3 + 2 h endurance training (60–75% of WR_LT_)
4	Strength endurance (gym) Upper body	Strength endurance (gym) Upper body	Strength endurance (gym) Upper body
5	T1 + 2 h endurance training (60–75% of WR_LT_)	T2 + 2 h endurance training (60–75% of WR_LT_)	T3 + 2 h endurance training (60–75% of WR_LT_)
6	3–4 h of endurance training 60–75% of WRLT with high-speed intervals (2 × 6 × 10 s-max)	3–4 h of endurance training 60–75% of WRLT with high-speed intervals (2 × 6 × 10 s-max)	3–4 h of endurance training 60–75% of WRLT with high-speed intervals (2 × 6 × 10 s-max)
7	Day off	Day off	Day off

Abbreviations: T1, training in the laboratory (15 min of warm-up (65–70% WR_LT_/WR_LThyp_), 100% WR_LT_/WR_LThyp_ for 30 min and 15 min of cool-down at 65–75% WR_LT_/WR_LThyp_); T2, training in the laboratory (15 min of warm-up (65–70% WR_LT_/WR_LThyp_), 100% WR_LT_/WR_LThyp_ for 35 min and 15 min of cool-down at 65–70% WR_LT_/WR_LThyp_); T3, training in the laboratory (15 min of warm-up (65–70% WR_LT_/WR_LThyp_), 100% WR_LT_/WR_LThyp_ for 40 min and 15 min of cool-down at 65–70% WR_LT_/WR_LThyp_).

**Table 2:** Diet components during the experiment.

Protein (g)	Fat (g)	Carbohydrates (g)	Caloric intake (kcal)
204 ± 4.2	124.9 ± 5.9	384.7 ± 8.6	3479.5 ± 36.2

**Table 3:** Atherogenic indices in cyclists before (S1) and after training (S2) in LH-TL, IHT, and control groups (N).

Variables	LH-TL		IHT		N	
	Before (S1)	After (S2)	Before (S1)	After (S2)	Before (S1)	After (S2)
AIP	0.046 ± 0.187	−0.115 * ± 0.181	0.132 ± 0.225	0.097 ± 0.227	−0.003 ± 0.221	−0.004 ± 0.268
CRI-I	2.788 ± 0.511	2.345 ** ± 0.403	2.822 ± 0.694	2.598 ± 0.556	2.512 ± 0.706	2.390 ± 0.699
CRI-II	1.545 ± 0.409	1.182 ** ± 0.350	1.470 ± 0.651	1.304 ± 0.447	1.294 ± 0.644	1.142 ± 0.564

Abbreviations: AIP, atherogenic index of plasma: log10(TG/HDL-C); CRI-I, Castelli’s risk index I: TC/HDL-C; CRI-II, Castelli’s risk index II: LDL-C/HDL-C. * *p* < 0.05; ** *p* < 0.01 before vs. after training.

**Table 4:** Changes (∆) in the lipid profile in cyclists following interventions after controlling for the effect of baseline levels.

Variables	LH-TL	IHT	N
∆TC(mg/dL)	−15.24 * ± 5.32	−0.79 ± 5.37	1.38 ± 5.68
∆LDL-C(mg/dL)	−14.45 ± 4.50	−2.65 ± 4.45	−7.10 ± 4.78
∆HDL-C(mg/dL)	5.47 ± 2.84	3.44 ± 2.82	5.76 ± 3.01
∆TG(mg/dL)	−22.31 * ± 6.48	3.54 ± 6.67	0.56 ± 6.88

Abbreviations: * *p* < 0.05 LH-TL vs. N. Covariates are evaluated at the following values: TC = 169.9, LDL-C = 87.6, HDL-C = 65.4, TG = 78.9.

**Table 5:** Changes (∆) in atherogenic indices in cyclists following interventions after controlling for the effect of baseline levels.

Variables	LH-TL	IHT	N
AIP	−0.16 # ± 0.05	−0.02 ± 0.05	−0.01 ± 0.05
CRI-I	−0.42 ± 0.11	−0.19 ± 0.11	−0.18 ± 0.11
CRI-II	−0.32 ± 0.09	−0.15 ± 0.09	−0.20 ± 0.09

Abbreviations: # *p* < 0.08 LH-TL vs. N. Covariates are evaluated at the following values: AIP = 0.058, CRI-I = 2.707, CRI-II = 1.436.

**Table 6:** Body mass and composition of cyclists before (S1) and after training (S2) in the LH-TL, IHT and N groups.

Variables	LH-TL		IHT		N	
	Before (S1)	After (S2)	Before (S1)	After (S2)	Before (S1)	After (S2)
BM (kg)	70.4 ± 7.1	69.6 ± 6.9	70.8 ± 9.4	70.5 ± 9.1	69.9 ± 5.9	70.1 ± 5.4
%FAT	6.9 ± 2.1	6.7 ± 1.6	10.4 ± 2.6	10.8 ± 2.9	7.3 ± 1.2	7.6 ± 1.2
FFM (kg)	65.5 ± 6.0	64.9 ± 6.2	63.5 ± 8.9	62.9 ± 9.3	64.4 ± 6.2	64.4 ± 5.8

Abbreviations: BM, body mass; %FAT, fat content; FFM, fat free mass.

## Data Availability

The data presented in this study are available on request from the corresponding author.
